# Comparison of Anterior and Posterior Surgical Approaches in Total Hip Arthroplasty: Effect on Self-Reported and Functional Outcomes

**DOI:** 10.3390/jcm14061935

**Published:** 2025-03-13

**Authors:** Clayton Foster, Songyuan Gu, Chase Dean, Craig Hogan, Michael Dayton

**Affiliations:** Department of Orthopedics, University of Colorado Anschutz Medical Campus, Aurora, CO 80045, USA; claytondfoster@gmail.com (C.F.); craig.hogan@cuanschutz.edu (C.H.); michael.dayton@cuanschutz.edu (M.D.)

**Keywords:** arthroplasty, replacement, hip, patient-reported outcomes, functional performance, direct anterior, posterior

## Abstract

**Background/Objectives:** Reported patient results after total hip arthroplasty (THA) have been described as a function of surgical approach. Such results have commonly been subjective. Though self-reported outcomes are of value and often utilized, inclusion of functional performance measures represents an objective measure to compare THA techniques. **Methods**: Patients that underwent primary THA surgery at our institution were grouped by surgical approach (Direct Anterior vs Posterior). Patient data were collected pre-operatively, as well as post-operatively at three and twelve months. Hip Dysfunction and Osteoarthritis Outcome Score (HOOS JR) was utilized, and function was assessed with the timed up and go test (TUGT), 4-m walk test (4MWT), and 30 s sit-to-stand (30STS) test. Unpaired T tests were used to compare mean results and differences between the groups. **Results**: Functional outcome scores were improved to a similar degree for both surgical approach groups at all the time points post-operatively. At 3 months, the TUGT was improved by 2.33 s for the posterior group, the 30STS was increased by 2.71 repetitions, and the 4MWT was increased by 1.23 s; the anterior group had 2.66 s, 2.49 repetition, and 1.18 s improvements in the three functional tests, respectively. At 12 months, the posterior group had improvements of 2.86 s, 3.99 repetition, and 1.19 s, while the anterior group had improvements of 3.15 s, 3.83 repetition, and 1.23 s, respectively. No clinical and statistical significant differences in surgical approach were noted in these measures. In contrast, the anterior group showed a statistically significant but not clinically significant improvement in self-reported HOOS JR scores compared to the posterior group at the 3-month post-operative mark (*p* = 0.045). **Conclusions**: This study suggests both anterior and posterior surgical approaches to total hip arthroplasty yield equivalent functional results at 3 months and one year post-operatively, while the anterior approach demonstrates more improved patient satisfaction than the posterior approach at the 3-month post-operative assessment.

## 1. Introduction

Total hip arthroplasty (THA) has witnessed a significant volume increase in recent decades [1]. In the United States alone it has increased by a factor of three from 2000 to 2010 [2]. The increased prevalence of primary THA is due to an increase in mean population age, as well as the success of THA in younger, active patients looking to decrease pain and improve function [3].

There is ongoing interest in varying surgical approaches, based on experience, exposure, and teaching. To a relative newcomer, the direct anterior (DA) approach is an intermuscular approach with an internervous plane and is described as causing less muscular disruption than the posterior approach, which retracts the gluteus maximus and the short external rotators [4,5]. Patients undergoing THA via the DA approach often omit range of motion restrictions that can be post-operatively imposed with posterior approach. Studies have demonstrated that the dislocation rate is lower in patients undergoing the DA approach compared to those undergoing the posterior approach. Clinical significance of this, however, is indeterminate depending upon adherence to recommended posterior hip precautions [6,7,8,9]. The DA approach has also been associated with a faster subjective recovery in early post-operative weeks; however, advantages have been inconsistent beyond three months post-operatively [10,11,12]. The anterior approach is not without disadvantage as well. Studies have reported the DA approach to be associated with increased operative time as well as a higher rate of peri-operative complications, including trochanteric fracture, femoral cortical perforation, lateral femoral cutaneous nerve neuropathy, and post-operative wound complications in patients with a higher BMI [13,14,15,16]. The learning curve for performing a THA through the direct anterior approach has been noted, with the literature suggesting that surgeons require 20–50 cases to avoid elevated complication rates [17,18].

Clinical studies comparing outcomes in total hip arthroplasty conventionally use patient self-reported outcomes as a primary measure for comparison of success. While self-reported outcomes provide useful perspective and numerical data, they are subject to patient recall bias. In contrast, quantitatively measuring functional outcomes provides a more objective approach to assessing post-operative outcomes for THA. As noted in previous studies regarding total joint arthroplasty, objectively measured functional outcomes often do not align with patients’ self-reported outcomes [19]. In the office setting, three simple and objective functional tests commonly used are the timed up and go (TUG) test, the 30-s sit-to-stand (30STS) test, and the 4-m walk test (4MWT). These tests have consistency and test–retest reliability in post-operative THA patients, making them reliable indicators of function [20,21,22,23].

The main goal of this study was to evaluate whether the posterior and direct anterior approaches to THA differ in functional performance outcomes. The secondary goal was to assess peri-operative parameters, post-operative complications, and patient-reported outcomes. We hypothesize that patients undergoing THA via the anterior and posterior approaches will have comparable post-operative self-reported and objective functional measures at 3 and 12 months following the procedure.

## 2. Materials and Methods

### 2.1. Study Design

As a retrospective cohort study, this included patients who underwent primary THA at this institution between April 2015 and December 2018. Two fellowship trained senior adult reconstruction orthopedic surgeons performed all surgeries at a single institution. Surgeon preference and patient considerations dictated the surgical approach. One surgeon (CAH) performed most anterior approaches, and most posterior approaches were performed by the other surgeon (MRD).

This study was approved by the affiliated Institutional Review Board. Inclusion criteria for the cohort were patients undergoing primary total hip arthroplasty in the designated period, by anterior or posterior approach, aged 18 to 90 years. Exclusion criteria included THA through an approach other than anterior or posterior, revision THA, and unwillingness or inability to consent to study inclusion.

All included patients completed a pre-operative assessment within 4 weeks of the surgery date. This included the HOOS JR Score assessment, active and passive range of motion (ROM), TUG test, 30STS test, and 4MWT. After January 2016, all patient-reported assessments were transitioned from the HOOS form to the HOOS JR form. Prior HOOS assessment scores were converted to HOOS JR scores for purposes of uniformity. The HOOS JR form has been validated as an accurate outcome measure in THA [24]. The same assessments were performed again at 3 and 12 months post-operatively with all patients.

**Figure 1:**
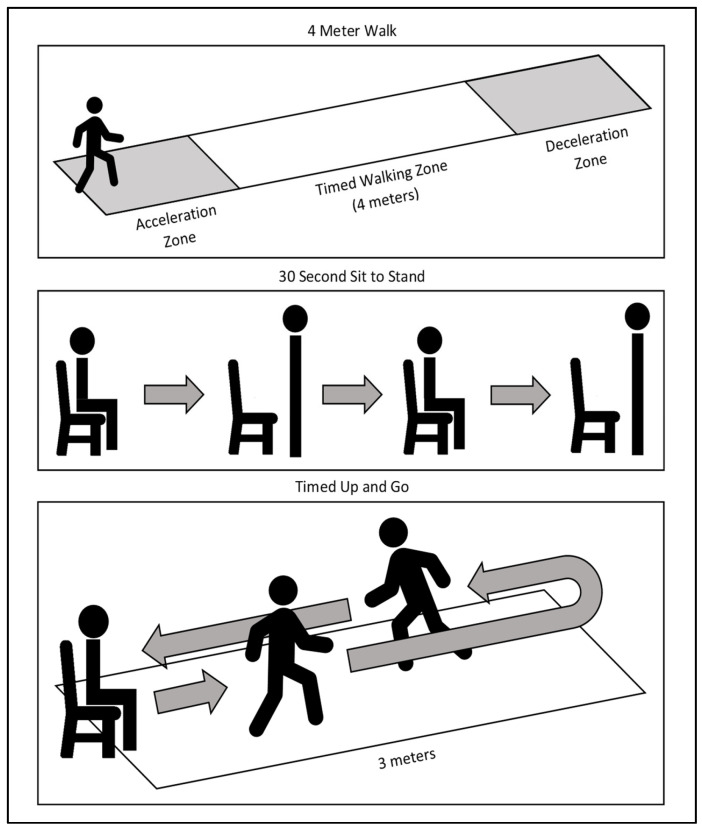
Functional tests illustrations; from top to bottom, the 4-m walk test, 30 s sit-to-stand test, and time up and go test are demonstrated.

The TUG test was performed with the patient starting in a seated position, as shown in Figure 1 [25]. The 30STS test was performed with the seated patient rising to standing and returning to sitting as many times as possible in a 30 s interval [21]. For the 4MWT, the amount of time it took the patient to travel the central four meters was recorded [20]. All test results were collected by one of three independent observers who were trained using the same standardized protocol.

All data were retrospectively collected via electronic medical record chart review (Epic Hyperdrive 100.2412.1.0). Pertinent demographic data were collected and are highlighted in Table 1. Collected peri-operative parameters included the surgical approach, time from incision to closure, estimated blood loss, and hospital stay duration (calculated by post-operative day of discharge). Adverse events that were recorded included fracture, dislocation, infection, and delayed wound healing.

### 2.2. Surgical Technique

For all patients, implants used were selected based on surgeon preference. Anesthesia administered was either general anesthetic or spinal anesthetic based on patient factors and anesthesiologist recommendations. All patients received an intra-operative local tissue injection prior to wound closure. This contained ropivacaine, morphine, ketorolac, and epinephrine, standard across both groups.

All anterior approach THAs were performed on a standard operating room table with bilateral lower extremities draped into the surgical field. Intra-operative fluoroscopy was used prior to incision for templating as well as at the time of acetabular and femoral component implantation to ensure satisfactory and safe implant positioning. The incision was 6 to 12 cm in length based upon patient factors, starting two fingerbreadths distal and lateral to the anterior superior iliac spine (ASIS). Deep dissection was taken medial to the tensor fascia lata (TFL) muscle belly and lateral to the indirect head of the rectus femoris. An anterior capsulotomy and partial capsulectomy were performed. The femoral head and neck were resected using a standard femoral neck osteotomy. After hip labral resection, the acetabulum was reamed, and the final acetabular component (Stryker, MI, USA) was inserted at an estimated 40° of inclination and 15–20° of anteversion. Zero to two screws (Stryker, MI, USA) were placed in the acetabular cup (Stryker, MI, USA). The leg of the bed was then flexed to place the hip in an extended, adducted, and externally rotated position for adequate proximal femoral exposure. The proximal femur was sequentially broached matching the native femoral anteversion. Final femoral components (Stryker, MI, USA) were placed after adequate stability and leg length measurement were obtained. Routine wound closure was performed without capsular repair.

For the posterior approach, the patient was placed in the lateral decubitus position on a standard operating table. A straight incision was made over posterolateral part of the greater trochanter, and the fibers of gluteus maximus muscle belly were retracted. A posterior hip capsulotomy was performed after piriformis and short external rotator tenotomies. The femoral head was posteriorly dislocated, and a femoral neck osteotomy was made. The labrum was excised, the acetabulum was reamed, and a cup (Exactech, Inc., Gainesville, FL, USA) was placed at 20–25 degrees of anteversion and 40° of inclination. Up to two posterior column acetabular screw(s) (Exactech, Inc., Gainesville, FL, USA) were placed. The femur was then broached in a standard fashion and final implants (Exactech, Inc., Gainesville, FL, USA) were trialed for stability and leg length. The wound was closed with posterior capsule, piriformis, and short external tendon repairs.

### 2.3. Post-Operative Protocol

All patients were admitted to the orthopedic inpatient floor post-operatively. The anterior group did not have any formal hip precautions. The posterior group had routine posterior hip precautions as follows: avoid hip flexion past 90°, avoid hip adduction or internal rotation beyond neutral. Patients were either mobilized on the day of surgery or the following morning with a physical and occupational therapist. More specifically, all total hip arthroplasty patients received physical therapy (PT) assistance on the day of surgery for early mobility. Full weight bearing was encouraged, and differences in activity were dictated by the surgical approach. While direct anterior approach hip patients require no restrictions, posterior approach hip patients necessitate posterior precautions in the form of avoidance of hip flexion > 90 degrees, concurrent hip flexion and internal rotation, and prohibition of crossing the legs. Hip abductor pillows are recommended for the posterior approach. Posterior hip precautions are discontinued at 6 weeks post-operatively. Patient needs for therapy are individualized; outpatient therapy is encouraged and facilitated beginning at 10–14 days, while some patients prefer to delay outpatient therapy for 6 weeks. Some patients experience home based physical therapy upon discharge, but due to variations in locale and insurance this is inconsistent. All patients consistently receive exercises upon discharge to allow for a self-guided home exercise program. Patients were required to stay a minimum of one night and were discharged to home or a post-acute advanced rehabilitation facility when medically and functionally stable per the therapy team recommendations.

### 2.4. Statistical Analysis

Patient demographic data were compared between the anterior and posterior groups using a chi-squared test or a two-sample *t*-test (Excel, 16.0). A test for normal variance (Excel, 16.0) was performed and satisfied for both groups. The pre-operative and three-month post-operative, as well as the twelve-month post-operative, objective measurements were compared between and within each group using a two tailed, two sample *t*-test (Excel, 16.0). The pre-operative, three-month post-operative, and twelve-month post-operative objective measurements were compared within each group using a two-tailed, two-sample *t*-test (Excel, 16.0). Two-sample *t*-tests (Excel, 16.0) were used to evaluate the significance of changes in measured values from the pre-operative baseline to the 3- and 12-month post-operative assessments for HOOS JR, the TUG test, the 30STS test, and the 4MWT between groups. The risk ratio (Excel, 16.0) was calculated to assess adverse event frequency and compared for significance using a chi-squared test (Excel, 16.0). For all parameters, statistical significance was defined as a *p*-value of less than 0.05.

## 3. Results

### General Findings

From April 2015 to December 2018, 850 consecutive patients underwent primary THA with one of two surgeons. Of these, seventy-one patients were excluded for having THA through an anterolateral approach. Additionally, one patient each was excluded due to primary diagnoses of septic hip and arthrodesis, respectively. By the end of the process, the anterior group included 400 patients, while the posterior group included 377 patients. At 3 months post-operative, 192 (48%) of the anterior group and 259 (69%) of the posterior group were available for follow up data. At twelve-month post-operative, 73 (18%) of the anterior group and 85 (22%) of the posterior group were available for follow up data. One surgeon (CAH) was responsible for 392 THAs, with 373 anterior and 19 posterior approaches. The second surgeon (MRD) performed 385 THAs, with 27 anterior and 358 posterior approaches.

The two groups showed no differences in respect to patient demographics (Table 1). The anterior group diagnoses included osteoarthritis (358), avascular necrosis (32), trauma (3), dysplasia (3), rheumatoid arthritis (2), psoriatic arthritis (1), and hemophilic arthritis (1). The posterior group diagnoses included osteoarthritis (353), avascular necrosis (16), dysplasia (5), rheumatoid arthritis (2), and trauma (1).

**Table 1:** Patient demographics.

	Anterior THA Average	Posterior THA Average	*p* Value
Age	62.3	60.9	0.904
Gender (Male)	181	188	0.677
Gender (Female)	219	189	0.151
Diabetes Mellitus (%)	7.50	10.9	0.440
History of DVT (%)	6.75	7.43	0.865
Smoker	11.3	9.76	0.745
BMI	27.1	29.3	0.769

There were no significant differences in operative time (anterior: 87.6 (49–153) min, posterior: 89.4 (41–287) min; *p* = 0.86), estimated blood loss (anterior: 299.7 (100–1000) mL, posterior: 271.4 (100–1500) mL; *p* = 0.25), or day of discharge (anterior post-op day: 1.9 (0–13), posterior post-op day: 2.7 (0–10); *p* = 0.70) (Table 2).

The anterior and posterior groups both showed significant improvement in HOOS JR scores from the pre-operative score at both 3- and 12-months post-operatively. (Anterior: pre-op 12.46 (0–24), 3-month 3.77 (0–20), *p* < 0.001; 12-month 3.63 (0–21), *p* < 0.001. Posterior: pre-op 12.50 (0–24), post-op 4.68 (0–23), *p* < 0.001; 12-month 4.02 (0–23), *p* < 0.001). When comparing the anterior and posterior groups, the HOOS JR score improved significantly more in the anterior group at three-months post-operatively (anterior: 8.59 (−6–21), posterior: 7.65 (−7–23), *p* = 0.045). However, the difference in HOOS JR score improvement between the anterior and posterior groups was not significant at 12 months post-operatively (anterior: 8.52 (−4–19), posterior: 8.51 (−8–20), *p* = 0.99).

There was significant improvement from pre-operative scores to 3- and 12-months post-operatively in all objective functional tests for both the anterior and posterior groups (all *p*-values 0.006 or less). Table 3 compares the change in scores between the anterior and posterior groups at three- and twelve-months post-operatively. There were no differences in the improvement achieved by the anterior group when compared to the posterior group for the TUG test, 30STS test, or 4MWT at either time point (all *p*-values 0.5 or greater).

The difference in adverse event ratios between the two groups was not significant; although, there was a trend toward increased infections in the posterior group (anterior: 0.50%, posterior: 2.11%, *p* = 0.06) (Table 4).

## 4. Discussion

This study found that patients achieved clinically and statistically similar functional performance outcomes at 3 and 12 months post-operatively in THAs performed using either the anterior or posterior approach. The data support the study hypothesis and suggests that THA performed through either the anterior or posterior approach restores functional capacity to a similar degree at both the 3-month and one-year post-operative time points. Patient-reported outcomes, as measured by the HOOS JR score, showed a statistically significant improvement in the anterior group compared to the posterior group at 3 months post-operatively. However, this difference was not clinically significant and became statistically insignificant at the 12-month post-operative time point. By 12 months, HOOS JR scores had improved to a similar extent in both the anterior and posterior groups (anterior: 8.52, posterior: 8.51, *p* = 0.99). This finding is consistent with the previously published literature suggesting patients with an anterior approach have a faster perceived recovery in the initial weeks after surgery. Significant differences, however, have failed to persist beyond 3-months post-operatively [11,12,26,27]. This suggests that the choice of surgical approach should be guided by the surgeon’s expertise and experience, along with patient-specific preferences, physiological factors, and functional baselines.

Primary outcome measures in total joint arthroplasty studies traditionally focus on patient-reported outcomes. Barrett et al. randomized patients to a direct anterior approach or posterior approach and compared outcomes at six weeks as well as at 3, 6, and 12 months post-operatively. A randomized controlled trial conducted by Barrett et al. compared patient-reported outcomes between the anterior and posterior approaches at 6 weeks, as well as at 3, 6, and 12 months post-operatively. The authors found a significantly higher Harris Hip Score (HHS) and HOOS in patients with anterior approach at both six-weeks and three-months post-operatively, but no significant differences existed beyond that point [12]. An additional meta-analysis looking at a combination of patient-reported and functional outcomes found significantly improved early pain scores in only 4 of the 17 included studies, and it also found a significantly shorter length of stay and lower dislocation rate for the anterior approach [7].

A variety of methods have been used to objectively compare functional performance outcomes following anterior and posterior approaches to THA. Taunton et al. compared time to cessation of using walking aids and found that anterior approach patients voluntarily discontinued assistive devices earlier than the posterior counterparts by six days [27]. In contrast, Rathod et al. reported no significant difference in gait analysis at 6- or 12-months post-operatively; although, they noted patients had significantly improved internal and external rotation of the hip following THA using the anterior approach [28]. Winther et al. assessed muscle strength in three approaches (anterior, posterior, direct lateral) and found stronger abduction strength in the posterior group at only six-weeks post-operatively, but no differences between any approach group at three-months with abduction strength or leg press [29]. The improvement in early patient-reported outcomes following the direct anterior approach to THA may result from several factors. Because the posterior approach requires disruption of the short external rotators to access the hip joint, patients are given instructions to limit flexion, adduction, and internal rotation post-operatively [30,31]. This approach contrasts with the intermuscular plane of the direct anterior approach which may allow patients to be unrestricted in their range of motion immediately post-operatively, potentially leading to an increased perception of improvement [30,31]. Additionally, the muscular disruption (of both gluteus maximus and the short external rotators) involved in the posterior approach may contribute to elevated pain immediately following the operation, as well as discomfort during sitting, reflected in self-reported outcome scores during the early post-operative period [30,31]. Although the analysis demonstrated a statistically significant increase in HOOS JR scores for patients who underwent the direct anterior (DA) approach compared to the posterior approach at the 3-month post-operative follow-up, this increase was not clinically significant. According to a study by Lyman et al., the minimum clinically important difference (MCID) for the HOOS JR score ranges from 7 to 36 [32,33]. In this study, the mean change in HOOS JR score at the 3-month post-operative period was 8.59 for the anterior approach group and 7.65 for the posterior approach group. While both exceeded the MCID threshold, the difference between the two was not clinically meaningful. These findings are consistent with the existing literature, indicating no clear evidence of superiority in patient-reported or functional outcomes between the direct anterior and posterior approaches [31,32,33].

The TUG test, 30STS test, and 4MWT have been validated as effective measures of objective patient function in total joint arthroplasty. Further, these functional tests are easy to perform and are reproducible. This is the only known study to primarily focus on these three objective functional measures to compare results in the anterior and posterior approaches to THA. Christensen et al. focused on six-week post-operative results in a total of 51 patients with a single surgeon and found no significant differences in the TUG test, chair rising force, or stair descent impact force. The authors did see improved pain scores in the anterior group, but no difference in any other patient-reported outcomes [34]. Rodriguez et al. similarly performed a meta-analysis looking at various patient-reported outcome measures, as well as functional outcomes using the TUG test, and found improved TUG scores for the anterior group at two-weeks post-operatively, but no significant differences existed in any measure beyond that time point [11]. Cheng et al. recently performed a prospective randomized study with 72 total patients comparing outcomes in anterior versus posterior THA at two-, six-, and twelve-weeks post-operatively. The authors in this case used a 10-m walk test and found no significant differences at any time point. They also reported no differences in patient-reported function (Western Ontario McMasters Arthritis Index and Oxford Hip Score) or quality of life score (EuroQol five dimensions questionnaire) at any time point [35].

An increased rate of trochanteric fractures is an observed and reported risk with the anterior approach, especially early in a surgeon’s adoption of this technique [8,15]. This study did not demonstrate any significant difference in the rate of trochanteric fractures between the two approaches (anterior: 2.0%, posterior: 1.06%, *p* = 0.25); however, it is likely that this risk was largely mitigated because the majority of the anterior approaches were performed by a single experienced surgeon. Similarly, previously published studies have reported increased operative time for the anterior approach compared to the posterior approach [36], but the absence of any difference here suggests that this difference may be overcome by surgeon experience. Additionally, because this study was performed at an academic institution and the patients were not randomly assigned to an approach, it is possible that patient factors and associated time spent teaching may be responsible for prolonging the average operative time of any specific approach. This is recognized as a potential weakness to the current study.

Previously published studies have demonstrated an increase in both the length of hospital stay and rate of post-operative infection for the anterior approach, particularly in obese patients [7,16,37]. The results of this study failed to achieve significance in support of this data, with infection risk demonstrating a trend in favor of the anterior approach (anterior: 0.50%, posterior: 2.11%, *p* = 0.06). However, these numbers fail to account for other comorbid conditions that may have contributed to increased length of hospital stay, thus allowing selection bias. Additionally, given the presence of the literature demonstrating an increased risk of infection following the anterior approach to THA, patients who were considered to have an elevated risk of infection were assigned to the posterior approach. This study also found no significant difference in dislocation rate or revision rate between the two approaches. Several large volume cohort studies, including those by Maratt et al. and Tripuraneni et al., have had similar findings [8,9]. Sheth et al. reviewed a large series of patients and also found no significant difference in revision rate; although, the authors did find a lower dislocation rate in the anterior approach compared to the posterior approach (anterior: 0.8%, posterior: 1.4%, *p* = 0.017) [6]. It is possible that the actual risk of dislocation is lower in the anterior approach to THA than in the posterior approach given the path of each approach. However, the clinical significance of this risk may be mitigated in the presence of adherence to range of motion limitations following posterior approach.

The current literature indicates that active smoking, obesity, pre-operative transfusion, and steroid use are all associated with an increased risk of surgical site infection and other post-operative complications [38,39,40,41,42,43,44,45,46]. Additionally, factors such as a history of tobacco use, lumbar spine disease, prolonged surgical and anesthesia duration, younger patient age (<45 years), and low surgeon-specific operative volume for hip arthroplasty have been shown to elevate the risk of post-operative nerve injury [41]. Revision hip arthroplasty significantly increases the risk of periprosthetic femoral fractures, whereas gender and the use of a cemented stem do not appear to have a significant impact [45]. Furthermore, discharge to a skilled nursing facility, lower surgical volume, and a high BMI have been identified as key risk factors for readmission at both 30 and 90 days following total hip arthroplasty (THA) [38,43]. Meta-analyses have also highlighted high ASA class, heart failure, diabetes, liver disease, alcohol consumption, depression, urinary tract infections, and deep vein thrombosis as factors positively correlated with increased 90-day readmissions following total joint replacement (TJR) [47]. More specifically, logistic regression analysis for both hip and knee arthroplasty has identified older age, revision surgery, bilateral procedures, general anesthesia, elevated glucose levels, a higher Charlson Comorbidity Index (CCI), and increased estimated blood loss (EBL) as significant predictors of unplanned ICU admission [43]. Additionally, smoking, a BMI > 30 kg/m^2^, diabetes, depression, steroid use, and frailty have been associated with an increased long-term risk of developing periprosthetic joint infections (PJI) [45,48]. For THA specifically, reduced pre-operative hemoglobin has been identified as an additional risk factor, while obesity, coronary artery disease (CAD), and pulmonary hypertension are independent risk factors for deep PJI in patients undergoing primary THA [45,48]. Based on the demographics of this study, the average BMI was relatively low (<30), with a mean of 27.1 in the anterior approach group and 29.3 in the posterior approach group. As no significant difference in BMI was observed between the two groups, it is not anticipated to be a major confounding factor affecting surgical outcomes. Additionally, age, gender, prior diabetes diagnosis, history of deep vein thrombosis (DVT), and smoking history were similarly distributed between the two groups without statistically significant differences. Therefore, these variables are not expected to be significant confounders that could skew the analysis. In addition, surgeon experience and case volume have been directly correlated with patient-reported outcomes, as well as peri-operative and post-operative complication rates [37,49]. The existence of a learning curve for the anterior approach in THA was confirmed by Foissey et al., while Hartford et al. reported a significant reduction in complication rates after the first 100 cases [37,49]. In this study, both surgeons are highly experienced, fellowship-trained specialists in adult arthroplasty, with case volumes far exceeding 100 procedures for their respective approaches. Therefore, surgeon experience is expected to have minimal impact on outcomes and complications, making it less likely to serve as a confounding factor in this analysis.

In this study, spinal anesthesia was utilized as an anesthetic option due to its proven effectiveness and safety profile in total hip arthroplasty procedures. A recent meta-analysis by Memtsoudis et al. recommends the use of neuraxial blockade over general anesthesia for primary hip arthroplasty [50]. Additionally, a meta-analysis by Messina et al. suggests that spinal anesthesia can be safely administered to elderly patients undergoing lower limb surgeries with minimal systemic side effects, including hemodynamic disturbances [51]. To maximize patient safety and minimize systemic side effects, pre-operative patient selection, intra-operative hemodynamic monitoring, and protocols for managing undesired hemodynamic disturbances were implemented at our institute for all THA procedures. Therefore, the choice of anesthesia is not considered a factor affecting outcomes for THA patients undergoing either approach.

This study exhibits several limitations. Firstly, as a retrospective cohort analysis, it is subject to selection bias and confounding variables. To minimize selection bias, we have defined and followed strict inclusion and exclusion criteria for all eligible patients during the defined period. Patient characteristics were not matched during analysis, potentially introducing confounding factors such as variations in surgical teams, patient demographics and comorbidities. Additionally, both study arms experienced a low follow-up rate, attributable in part to the tertiary care center location, requiring patients to travel long distances. More specifically, as a high-volume tertiary medical center, many of our patients are referred from across the region to receive specialized interventions such as hip arthroplasty. A significant number of them live far away or in remote areas, making it difficult for them to return for follow-up appointments. As a result, some patients opt to follow up with their initial referring provider or seek care from other providers closer to home. This referral pattern likely contributes to our high loss to follow-up at one year post-operatively. In addition, the absence of proactive follow-up reminders may have contributed to this issue. At the time of data collection several years ago, an automated reminder system for follow-up appointments was not in place. Instead, follow-up appointments were solely scheduled by patients, which may have contributed to the low follow-up rate. Furthermore, patients not seen within three weeks of the designated post-operative timeframe (3 or 12 months) did not undergo functional assessments, potentially impacting outcome evaluation. Notably, the sample size at the 12-month follow-up was limited, resulting in low statistical power (0.5895) with an alpha of 0.05 and a Cohen’s D of 0.35. This limitation compromises the accuracy of comparisons between pre-operative and 12-month post-operative outcomes and restricts the generalizability of the long-term findings. To maintain the robustness of the analysis with the available data and to properly account for missing data, patients lost to follow-up at either 3 months or one year were included in the analysis up to the point at which they were lost. For example, if a patient was lost to follow-up at one year, their pre-operative baseline and 3-month post-operative data were included in the analysis. Similarly, if a patient was lost to follow-up at 3 months, only their pre-operative baseline data were used. To address the limitations of the current study, future research should focus on a multicenter, prospective, randomized controlled trial with a minimum follow-up period of five years, incorporating an automated multimodal follow-up reminder system for patients. In addition to the HOOS JR score, subsequent studies should include the full HOOS score, Harris Hip Score (HHS), and a single-item Patient Acceptable Symptom State (PASS) survey to assess clinically meaningful differences more comprehensively in patient-reported outcomes [32,33]. Specific tests and imaging modalities could also be incorporated to enhance the evaluation of muscle damage and rehabilitation progress at various post-operative time points across different surgical approaches. For instance, pre-operative and post-operative CT scans could be utilized to assess muscle volume and CT density [52,53]. Handheld dynamometry could provide quantitative measurements of specific muscle group strength, while gait analysis could be employed to evaluate overall biomechanics and identify muscle deficits [52,53]. Furthermore, expanding the complication analysis to include neuropraxia, post-operative periprosthetic fractures, in-hospital complications, pneumonia, urinary tract infections (UTIs), and deep vein thrombosis/pulmonary embolism (DVT/PE) is recommended. Outcome measures should also be broadened to evaluate the total cost of the procedure, long-term joint survival rates, and overall patient survival rates.

## 5. Conclusions

This retrospective cohort study revealed that both the direct anterior and traditional posterior approaches to total hip arthroplasty yield identical functional performance results up to one year post-operatively. While the anterior approach may offer a more subjectively appealing post-operative recovery rate up to 3 months, it may be associated with disadvantages, including increased risk of trochanteric fractures and higher infection rates in obese populations. The present study demonstrated no statistical or clinical differences in restoring function or rates of complications among THA patients undergoing primary anterior versus posterior surgery. Given the comparable functional outcomes observed between the anterior and posterior approaches, it is recommended that surgeons engage patients in shared decision-making regarding the selection of the most suitable surgical approach. This process should consider the surgeon’s expertise and preferences, patient anatomy, and ensuring alignment with the patient’s individual needs and goals.

## Figures and Tables

**Table 2:** Peri-operative data.

	Anterior THA Average	Posterior THA Average	*p* Value (Chi^2^ test)
Operation Time (min)	87.6	89.4	0.86
Estimated blood loss (mL)	299.7	271.4	0.25
Length of hospital stay (days)	1.9	2.7	0.70

**Table 3:** Patient-reported and functional-outcome measures.

Changes at 3 Months Post-Op			
	Anterior THA Average	Posterior THA Average	*p* Value
HOOS JR Score	8.59	7.65	0.045
Time up and go (s)	2.66	2.33	0.519
30 s sit-to-stand (# of reps)	2.49	2.71	0.638
4-m walk test (s)	1.18	1.23	0.820
**Changes at 12 Months Post-Op**			
	**Anterior THA** **Average**	**Posterior THA** **Average**	***p* Value**
HOOS JR Score	8.52	8.51	0.99
Time up and go (s)	3.15	2.86	0.71
30 s sit-to-stand (# of reps)	3.83	3.99	0.85
4-m walk test (s)	1.23	1.19	0.91

**Table 4:** Adverse events summary.

	Anterior THA (Total, Average %)	Posterior THA (Total, Average %)	*p* Value
Trochanteric Fracture (#cases)	8 (400, 2.00%)	4 (379, 1.06%)	0.25
Post-Op Acetabular Fracture (#cases)	0 (400, 0.00%)	1 (379, 0.26%)	0.32
Post-Op Dislocation (#cases)	5 (400, 1.25%)	10 (379, 2.64%)	0.20
Post-Op Infection (#cases)	2 (400, 0.50%)	8 (379, 2.11%)	0.06
Post-Op wound complication (#cases)	3 (400, 0.75%)	4 (379,1.06%)	0.71
Revision (#cases)	7 (400, 1.75%)	13 (379, 3.43%)	0.14

## Data Availability

The data presented in this study are available on request from the corresponding author due to privacy and HIPAA restrictions.

## Abbreviations

The following abbreviations are used in this manuscript:

THA	total hip arthroplasty
TUGT	timed up and go test
30STS	30 s sit-to-stand test
4MWT	4-m walk test
DA	direct anterior
ROM	range of motion
TFL	tensor fascia lata
ASIS	anterior superior iliac spine
HHS	Harris Hip Score
